# Long-Range Chromosome Organization in *E. coli*: A Site-Specific System Isolates the Ter Macrodomain

**DOI:** 10.1371/journal.pgen.1002672

**Published:** 2012-04-19

**Authors:** Axel Thiel, Michèle Valens, Isabelle Vallet-Gely, Olivier Espéli, Frédéric Boccard

**Affiliations:** 1 CNRS, Centre de Génétique Moléculaire, UPR3404, Gif-sur-Yvette, France; 2 Université Paris-Sud, Orsay, France; Uppsala University, Sweden

## Abstract

The organization of the *Escherichia coli* chromosome into a ring composed of four macrodomains and two less-structured regions influences the segregation of sister chromatids and the mobility of chromosomal DNA. The structuring of the terminus region (Ter) into a macrodomain relies on the interaction of the protein MatP with a 13-bp target called *matS* repeated 23 times in the 800-kb-long domain. Here, by using a new method that allows the transposition of any chromosomal segment at a defined position on the genetic map, we reveal a site-specific system that restricts to the Ter region a constraining process that reduces DNA mobility and delays loci segregation. Remarkably, the constraining process is regulated during the cell cycle and occurs only when the Ter MD is associated with the division machinery at mid-cell. The change of DNA properties does not rely on the presence of a *trans*-acting mechanism but rather involves a *cis*-effect acting at a long distance from the Ter region. Two specific 12-bp sequences located in the flanking Left and Right macrodomains and a newly identified protein designated YfbV conserved with MatP through evolution are required to impede the spreading of the constraining process to the rest of the chromosome. Our results unravel a site-specific system required to restrict to the Ter region the consequences of anchoring the Ter MD to the division machinery.

## Author Summary

The large size of genomes compared to cell dimensions imposes an extensive compaction of chromosomes compatible with various processes of DNA metabolism, such as gene expression or segregation of the genetic information. Most bacterial genomes are circular molecules, and DNA replication proceeds bidirectionally from a single origin to an opposite region where replication forks meet. In the bacteria *Escherichia coli*, the long-range organization of the chromosome relies on the presence of mechanisms that structure large regions called macrodomains. The macrodomain containing the terminus of replication is structured by a specific organization system involving the binding of the protein MatP to 23 matS sites scattered over the 800-kb-long Ter region. In this report, we describe a site-specific insulation system that restricts to the Ter region the consequences of the mechanism structuring the Ter macrodomain. We identified two 12-bp sequences flanking the Ter macrodomain and one protein that are required to isolate the Ter region from the other parts of the chromosome.

## Introduction

The large size of genomes compared to cell dimensions imposes an extensive compaction of chromosomes compatible with various processes of DNA metabolism such as gene expression, replication and segregation of the genetic information. The organized bacterial chromosome, named the nucleoid, is a compact structure that presents a particular orientation inside the cell preserving the linear order of genes on DNA [1]. Although our understanding of the global organization of the bacterial chromosome is still limited, different levels of organization have been identified. At the molecular level, binding of a number of proteins including nucleoid-associated proteins (NAPs), Structural Maintenance of Chromosomes (SMC) proteins, transcriptional regulators, chromosome organizers or RNA polymerase to the DNA molecule results in the formation of a bacterial chromatin with different properties according to the identity of proteins bound [2]–[4]. The excess of negative supercoils generates the formation of plectonemes that impose a secondary structure to the DNA molecule [5]. The size of such structures has been estimated to range in size from 10 kb to 100 kb depending on the studies [6]–[8].

At a higher level, there are several levels of organization required to fully condense the *E. coli* chromosome. Recent studies have implicated the H-NS protein in the formation of two clusters per chromosome in which H-NS regulated genes are sequestered [9]. Using FISH and genetic approaches, a long range organization based on the existence of four insulated macrodomains (MD) and two less constrained regions called non-structured (NS) regions has been uncovered [10], [11]. MDs have been defined as large regions in which DNA interactions occurred preferentially and DNA interactions between the different MDs are highly restricted. In NS regions, DNA sites can interact with both flanking MDs [11]. The Ori MD contains *oriC* while the opposite Ter MD contains the replication terminus and the chromosome dimer resolution *dif* site. The Ter MD is flanked by the Left and Right MDs whereas the Ori MD is flanked by the two NS (NS^Right^ and NS^Left^) regions [11]. This organization influences the segregation of sister chromatids and the mobility of chromosomal DNA [12].

Structuring of the Ter MD relies on the binding of the MatP protein to a 13 bp motif called *matS* repeated 23 times in the 800 kb long domain. This protein accumulates in the cell as a discrete focus that colocalizes with the Ter MD. In the absence of MatP, DNA is less compacted, the mobility of markers is increased and segregation of the Ter MD occurs early in the cell cycle [13]. Moreover, recent studies showed that these two last aspects are also affected in a *zapB* mutant. ZapB, which is associated to the division machinery, stabilizes the Ter MD at mid-cell through a direct interaction with MatP (Espéli et al., submitted). Interestingly, bioinformatic analyses showed that *matS* sequences are present in different enterobacteria and *Vibrio* species [13] while MatP has been shown to belong to a group of proteins (including SeqA and MukBEF) exclusively identified in bacteria carrying Dam methyltransferase activity [14].

The molecular basis for structuring the other MDs remains to be characterized. However, repeated sequences analogous to *matS* have not been found in the other MDs and other models should be considered. In order to investigate the effect of the relative position of the MD on its properties, we designed a new method reminiscent of a cut-and-paste transposition event that allows the relocation of any chromosomal fragment at a defined position of the genetic map. Using this “transposition” technique, we generated a number of new chromosomal configurations. Very interestingly, we noticed that transposing the NS regions close to the Ter MD leads to a dramatic decrease in the mobility of fluorescent markers. We further identified two palindromic sequences called *tid*R and *tid*L (found in the Right and Left MDs, respectively) that are required in order for the NS regions to keep their specific properties. In the absence of these sequences, a constraining effect dependent upon the association of the Ter MD to the division machinery and acting in *cis* is detected at a very large distance, up to 750 kb of the Ter MD. Strikingly, we identified a protein, called YfbV, also exclusively found in bacteria possessing the Dam methylase which is required to prevent this constraining process of the NS regions and flanking Right and Left MDs.

Overall, this study shows that specific determinants (two palindromic sequences and a protein) are required in order to prevent a MatP dependent constraining effect to affect chromosome arms flanking the Ter MD, therefore “insulating” the Ter MD from other parts of the chromosome.

## Results

### The “Transposition” method: Displacement of chromosomal fragments without affecting gene orientation

In several studies, genomic rearrangements have been used to unveil the principles of chromosome organization [15]–[18]. In these cases, recombination between inverted recombining sites was used to promote genomic rearrangement. Unfortunately, this method perturbs several parameters at the same time: for example, inversion of the segment encompassing the Ori and Right MDs changes the relative position of the involved regions in Ori and Right MDs but also affects the orientation of more than one thousand genes including those in the intervening NS region. To limit the number of parameters affected by genetic rearrangements, we devised a new strategy reminiscent of a cut-and-paste transposition event that allows relocating any chromosomal fragment at a defined position of the genetic map without changing gene orientation. It relies upon the site-specific recombination Int system from bacteriophage λ. The Int integrase mediates phage integration in the chromosome by recombination between phage *att*P and bacterial *att*B attachment sites (integrative recombination) generating two hybrid sites, *att*L and *att*R. Recombination between *att*L and *att*R (excisive recombination) requires the additional presence of the Xis excisionase. We used directly repeated *attL* and *attR* sites flanked by the 5′and 3′ parts of *lacZ*, respectively [11], to delimitate the genomic fragments to relocate. Recombination between these two sites leads to the formation of two circles, one carrying *attB* in frame within a functional *lacZ* gene, and one carrying *att*P. An additional *attB* site (called *attB′*) was integrated at the target site. The *int* and *xis* genes were cloned under the control of a thermosensitive promoter [11].

**Figure 1:**
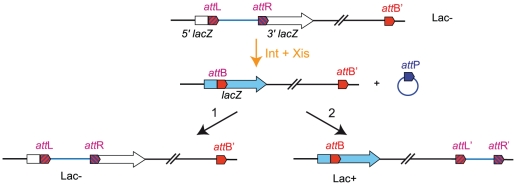
Chromosomal rearrangement by transposition. The three *att* sites are integrated in the chromosome in the same orientation. *att*L and *att*R sites are flanked by the 5′ and 3′ parts of *lacZ*, respectively [11]. An additional *att*B site (*att*B′) is inserted at a defined position. Excisive recombination promoted by Int+Xis results in the excision and circularization of the intervening segment carrying *att*P. This recombination event reconstitutes a functional *lacZ*. The excised molecule is not replicated and should be reintegrated for the viability of cells. Integration can occur in reconstituted *att*B (1) or in *att*B′ (2). Insertion in *att*B′ gives rise to Lac^+^ recombinants.

The transposition takes place in two steps (Figure 1). First, excisive recombination between *att*L and *att*R sites promoted by Int and Xis generates two circles: one carrying *att*P (the excised segment) and one carrying *att*B (the chromosome with a deletion of the excised segment). As the excised segment carries essential genes, its loss is fatal to the cell. Therefore, the second step of the transposition process consists in its reinsertion into the chromosome, either in the reconstituted *att*B fused to *lacZ* or in *att*B′ (giving rise to *att*L′ and *att*R′ sites) (Figure 1). The two events can be discriminated by the absence or presence of β-galactosidase activity. Conditions that provide a large amount of Xis and Int recombinases were applied to ensure that the excision of the segment between *att*R and *att*L occurs at a high frequency, greater than 95%. The level of lethality was greater than 90%, indicating that reinsertion of the excised segment occurred at low frequency. This is probably due to the presence of Xis that inhibits integrative recombination. However the amount of recombination was sufficient to obtain every transposition we tried. Insertion in the *att*B′ site and formation of the *att*L′ and *att*R′ sites can be detected by the appearance of blue clones on plates containing X-Gal and can be demonstrated at the DNA level by PCR analysis (Figure S1).

### Constraining of the NS regions is observed upon relocation next to the Ter MD

**Figure 2:**
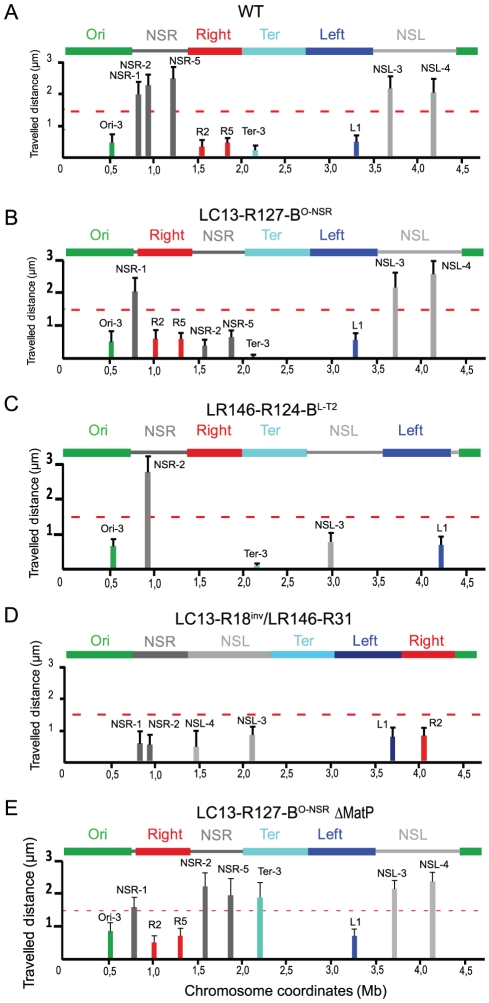
Travelled distance of different markers in strains with a wt or rearranged configurations. The mean value and the standard deviation calculated for 30 independent foci are indicated by columns. The x axis represents the chromosome genetic map; the positions of the loci are in Mb from *oriC*. The MDs and NS regions are indicated above the graph. (A) wt strain (markers Ori-3, NSR-1, NSR-2, NSR-5, Right-2 (R2), Right-5 (R5), Ter-3, Left-1 (L1), NSL-3 and NSL-4 are indicated from left to right). (B) Strain LC13-R127-B^O-NSR^ with a transposition of the Right MD between the Ori MD and the NS^Right^ region (markers Ori-3, NSR-1, Right-2 (R2), Right-5 (R5), NSR-2, NSR-5, Ter-3, Left-1 (L1), NSL-3, and NSL-4 are indicated from left to right). (C) Strain LR146-R124-B^L-T2^ with the NS^Left^ region transposed between the Ter and left MDs (markers Ori-3, NSR-2, Ter-3, NSL-3, and Left-1(L1) are indicated from left to right). (D) strain LC13-R18^inv^/LR146-R31 with the two NS regions located on the right replication arm (markers NSR-1, NSR-2, NSL-3, NSL-4, Left-1, Right-2) (E) strain LC13-R127-B^O-NSR^ Δ*matP* with a transposition of the Right MD between the Ori MD and the NS^Right^ region with a deletion of *matP* (markers Ori-3, NSR-1, Right-2 (R2), Right-5 (R5), NSR-2, NSR-5, Ter-3, Left-1 (L1), NSL-3 and NSL-4 are indicated from left to right).

Using fluorescent microscopy, we showed in a previous study that the dynamic behaviour of loci was different according to their belonging to a MD or a NS region. The system used derived from the bacteriophage P1 partition module and involved the ParB-GFP fusion protein interacting with *parS* sites inserted in the chromosome [19]. By time-lapse imaging during a 5 min period with a 10 sec interval, tracking of *parS* tags inserted in different regions of the chromosome and decorated by a fluorescent ParB-GFP fusion protein allowed to measure the travelled distance by each tag and its diffusion coefficient; it revealed that constraints on mobility are higher in MDs than in NS regions [12]. In order to investigate the effect of position on these different behaviours, travelled distances (Figure 2) and diffusion coefficients (Table S1) were measured in different strains (Table 1) for a number of loci (NSR-1, NSR-2, NSR-5, Right-2, Right-5, Ter-3, Left-2, Left-1, NSL-3, NSL-4, Ori-3; for their exact location, see Table S2) in different chromosomal configurations. Results are shown in Figure 2.

**Table 1:** Strains and plasmids.

Strain name					Reference
	Transposition coordinates			Configuration after transposition^a^	
	attL	attR	attB′		
LC13-R127 B^O-NSR^	1099533	651775	153248	Ori-Right-NS^R^-Ter-Left-NS^L^	This work
LR146-R124 B^L-T2^	3697780	2892861	1920110	Ori-NS^R^-Right-Ter-NS^L^-Left	This work
LR14-R127 B^O-NSR^	914188	651775	153248	Ori-Right^651-914^-NS^R^-Right^914-1135^-Ter-Left-NS^L^	This work
LC13-R17 B^O-NSR^	1099533	806540	153248	Ori-Right^806-1099^-NS^R^-Right^600-806^-Ter-Left-NS^L^	This work
LR132-R127 B^O-NSR^	993242	651775	153248	Ori-Right^651-993^-NS^R^-Right^993-1135^-Ter-Left-NS^L^	This work
LR128-R127 B^O-NSR^	1002340	651775	153248	Ori-Right^651-1002^-NS^R^-Right^1002-1135^-Ter-Left-NS^L^	This work
LR146-R124 B^L-T^	3697780	2892861	2202349	Ori-NS^R^-Right-Ter-Left^1920-2202^-NS^L^-Left^2202-2892^	This work

	Inversion coordinates		Configuration after inversion^b^	
	attL	attR		
LC13-R18	1099533	2879350	Ori-NS^R^-Right-Left-Ter-NS^L^	This work
LC13-R18^inv^/LR146-R31	3697780	602656	Ori-NS^R^-NS^L^-Ter-Left-Right	This work
LC13-R524	1099533	2050800	Ori-NS^R^-Right-Left^2050-1914^-Ter-Left^2050-2900^-NS^L^	This work

	Description	
FBG150	MG1655 Δ*lacIZ* Δ*att*B^λ^::*aadA*	[11]
Δrins1	FBG150 Δ(993250–1002300)::*frt-cat-frt*	This work
Δrins2	FBG150 Δ(993250–995200):: *frt-cat-frt*	This work
Δrins3	FBG150 Δ(995200–998414):: *frt-cat-frt*	This work
Δrins4	FBG150 Δ(998414–1002300):: *frt-cat-frt*	This work
Δrins5	FBG150 Δ(995250–996735):: *frt-cat-frt*	This work
Δrins6	FBG150 Δ(997713–1002300):: *frt-cat-frt*	This work
Δrins2.1	FBG150 Δ(993250–993750):: *frt-cat-frt*	This work
Δrins2.2	FBG150 Δ(993750–994250):: *frt-cat-frt*	This work
Δrins2.3	FBG150 Δ(994250–994750):: *frt-cat-frt*	This work
Δrins2.4	FBG150 Δ(994750–995211):: *frt-cat-frt*	This work
Δrins2.5 or ΔtidR	FBG150 Δ(993333–993344):: *frt-cat-frt*	This work
Δ*rins2 ykgC-ykgD*::rins2.1	Δrins2 *ykgC*-*ykgD*::(993250–993750)-*frt*-*arr2-frt*	This work
Δrins2 *ykgC-ykgD*::tidL	Δrins2 *ykgC*-*ykgD*::*tidL-arr2*	This work
Δ*tidR* ykgC-ykgD::tidR	Δrins2 *ykgC*-*ykgD*::*tidRarr2*	This work
Δ*tidR* ykgC-ykgD::tid^shuffled^	Δrins2 *ykgC*-*ykgD*::ctrl*^tidR^* –*arr2*	This work
FBG150 ΔyfbV	Δ(2410697–2411152):: *frt-cat-frt*	

Plasmid name	Description	
pALA2705	colE1 derivative carrying GFP-Δ30ParBP1	[19]
pCP20	pSC101^ts^ derivative carrying *cI857*-P_R_-*flp*	[27]
pTSA29-CXI	pSC101^ts^ derivative carrying *cI857*-P_R_-(*xis-int*)	[11]
pFH2973	colE1 derivative carrying *CFP-*Δ*30ParB* ^P1^/*YFP-*Δ*30ParB* ^T1^	[30]
pKD46	pSC101ts derivative carrying *γ*, *β*, *exo*	[27]

We first transposed most of the NS^Right^ region between the Ter and Right MDs (strain LC13-R127-B^O-NSR^). Such rearrangement had no dramatic effect on cell growth, cell and nucleoid aspects; a mild effect was detected in competition experiments, resulting in a 1 to 10 ratio in competition assays after 80 generations (Figure S2). Remarkably, mobility was specifically affected in the transposed NS^Right^ region (markers NSR-2 and NSR-5) whereas it was unchanged in other regions of the chromosome, including the NS^Left^ region (Figure 2B).

We next transposed most of the NS^Left^ region between the Ter and Left MDs (strain LR146-R124 B^L-T2^). Strikingly, the marker NSL-3 in the NS^Left^ region specifically showed a decreased mobility, whereas markers in other regions (including NSR-2 in the NS^Right^ region) were not affected (Figure 2C).

We also constructed a strain in which the two NS regions are positioned on the same replication arm of the chromosome (strain LC13-R18^inv^/LR146-R31 in Table 1). Strikingly, markers located in the two NS regions showed a decreased mobility (Figure 2D). This indicates that the constraining of the NS regions is not linked to their position relative to the Right or the Left MDs. Rather it may depend on proximity of the Ter MD.

Structuring of the Ter MD relies on the binding of the MatP protein to its target sequence *matS*. To determine whether the constraining process which originates from the Ter region requires its structuring into a MD, we analysed the changes in mobility of markers in cells devoid of MatP. We deleted the *matP* gene in the LC13-R127-B^O-NSR^ strain, and assayed the mobility of markers located in different regions of the chromosome (Figure 2E). Remarkably, markers located in the NS^Right^ region next to the Ter were mobile, in contrast to what is observed when MatP was present (see Figure 2B for comparison). The mobility of a Ter marker was also increased in a *matP* mutant, as expected, whereas the mobility of markers located in the Right and Left MD was not affected (Figure 2E). These results indicated that the constraining process associated to the Ter region required the MatP protein.

### Identification of insulation determinants *tidR* and *tidL*

**Figure 3:**
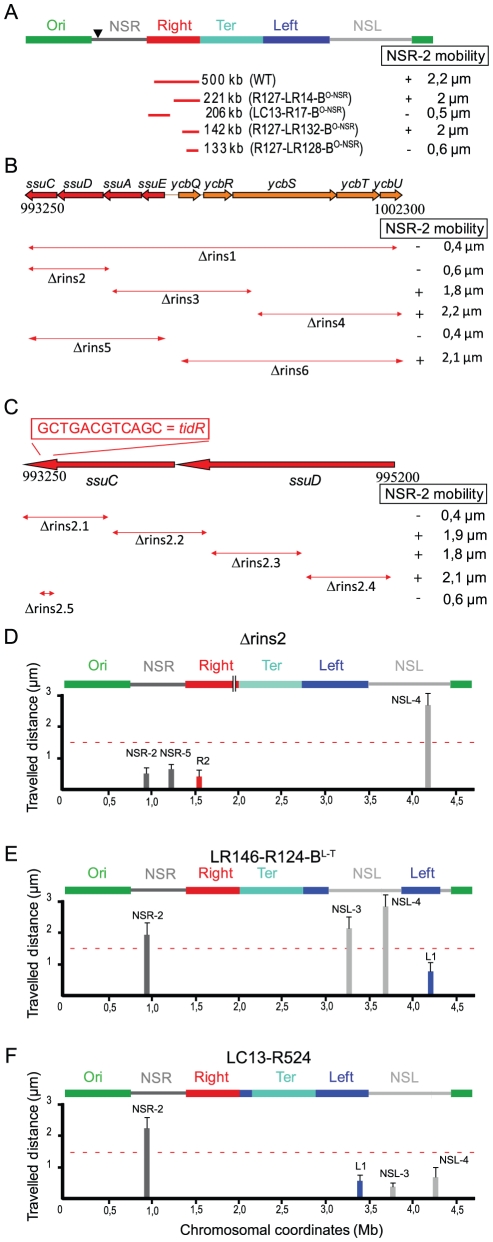
Identification of insulation determinants *tid*R and *tid*L. (A) Transposition of different fragments of Right MD between the Ori MD and the NS^Right^ region (indicated by an arrowhead). The remaining part of the Right MD separating the NS^Right^ region from the Ter MD is indicated by the red lines. The size of the remaining part is indicated in kb beside the name of the strain. Mobility of the marker NSR-2 is given and the presence of the insulation determinants is indicated by “+”. (B) Effect of deletions spanning the *ssuEADC-ycbQRSTU* region on NSR-2 mobility. The extent of the deletion is indicated below the map of the region (coordinates are indicated in bp). Mobility of the marker NSR-2 is given and the presence of the insulation determinants is indicated by “+”. (C) Effect of deletions spanning the *ssuCD* region on NSR-2 mobility. The extent of the deletion is indicated below the map of the region (coordinates are indicated in bp). Mobility of the marker NSR-2 is given and the presence of the insulation determinants is indicated by “+”. The position and sequence of *tid*R is indicated above the map. (D) Travelled distance of different markers in strain Δrins2 as indicated in Figure 2. The deletion of segment rins2 is schematized by a double line inside the Right MD (markers NSR-2, NSR-5, Right-2 (R2), NSL-4 are indicated from left to right). (E) Travelled distance of different markers in strain LR146-R124-B^L-T^ with a transposition of the NS^Left^ region transposed inside the Left MD (markers NSR-2, NSL-3, NSL-4, and Left-1 (L1) are indicated from left to right). The insulation determinants were present in the 290 kb of the Left MD proximal to the Ter MD. (F) Travelled distance of different markers in strain LC13-R524 with an inversion between sites located in the Right and Left MD (markers NSR-2, Left-1 (L1), NSL-3, and NSL-4 are indicated from left to right). No insulation determinants were present in the 830 kb of the Left MD distal to the Ter MD; they are predicted to be present in the 135 kb segment proximal to the Ter MD and that has been positioned between the Ter and Right MDs by the inversion event.

Experiments described above suggest that the Right and Left MDs may insulate the NS regions from a constraint linked to the Ter MD proximity. In order to test this hypothesis and investigate what regions of the Right MD was required to protect the NS^Right^ region, we moved various parts of the Right MD by transposition and tested the effect of remaining parts of the Right MD on the mobility of markers in the NS^Right^ region. Results are presented in Figure 3A. They showed that this mobility was not affected when the 221 kb region closest to the Ter MD (coordinates 914 kb to 1135 kb) remained between the Ter MD and the NS region (strain R127-LR14-B^O-NSR^) whereas the 206 kb region nearest from the NS^Right^ region (coordinates 600 kb to 806 kb) was not sufficient to prevent a decrease in mobility of the NS region (strain LC13-R17-B^O-NSR^). In this strain, the mobility of a marker located in this 206 kb segment of the Right MD was the same as in a wild type chromosomal configuration (data not shown). This indicated that the structuring of the Right MD by itself was not affected, and that it was not sufficient to protect the NS region. This also revealed that specific insulation determinants were present in the 221 kb segment adjacent to the Ter MD.

We further dissected what part of this 221 kb segment carried these determinants, using the same strategy. We showed that they were present in the 142 kb segment flanking the Ter MD (coordinates 993 kb from 1135 kb; strain R127-LR132-B^O-NSR^) but not in the 133 kb segment flanking the Ter MD (coordinates 1002 kb to 1135 kb; strain R127-LR128-B^O-NSR^ in Figure 3A). This demonstrated that insulation determinants were present in the 9 kb region located between coordinate 993 kb to 1002 kb, which encompasses 10 genes (*ssuCDAE-ycbQRSTU*) predicted as not essential for the viability of *E. coli*. Deletion of this 9 kb region was engineered (strain Δrins1) and we measured the mobility of different markers. A specific reduction of mobility was observed for the marker NSR-2 in the NS^Right^ region, confirming that insulation determinants are present in that 9 kb region.

Interestingly, these results showed that the constraining effect promoted by the Ter MD was also detectable in the native chromosome configuration provided that the insulating determinants were removed indicating that immediate proximity of the Ter MD was not required.

Deletions spanning the *ssuCDAE-ycbQRSTU* region were generated to map more precisely the insulation determinants and the effect of these deletions on the mobility of markers located in the NS^Right^ region was assayed. The first series of deletions indicated that determinants were present in the *ssuCD* region (Figure 3B). More precise deletions were subsequently generated and the results showed that only a 12 bp palindromic sequence (GCTGACGTCAGC) located in the 3′ end region of *ssuC* was required for the insulation phenomenon (Figure 3C). Indeed, deletion of these 12 bp induced a specific decrease in mobility of markers NSR-2 and NSR-5, whereas other markers (such as NSL-4 in the NS^Left^ region) were not affected (strain Δ*tid*R in Figure 3D). This sequence was called *tidR* for *t*er MD *i*nsulation *d*eterminant *R*ight. Its deletion had no important consequences for cell morphology and nucleoid distribution (Figure S3).

As described above, the constraining process seemed to be at work on both sides of the Ter MD. In order to map insulation determinants responsible for preventing a change of properties in the NS^Left^ region, a number of chromosomal rearrangements involving the Left MD and the NS^Left^ region were generated (strains LR146-R124-B^L-T^ and LC13-R524). Results showed that insulation determinants were present in the 282 kb segment flanking the Ter MD (strains LR146-R124-B^L-T^ in Figure 3E) and presumably present in the 135 kb segment flanking the Ter MD (strain LC13-R524 in Figure 3F and data not shown). A bioinformatic analysis of this 135 kb fragment allowed the identification of a 12 bp sequence that differed from *tid*R by a single nucleotide (GTTGACGTCAGC) that was called *tidL* for *t*er MD *i*nsulation *d*eterminant *L*eft. This sequence was located in the *tar* gene, encoding a methyl-accepting chemotaxis protein. The effect of *tidL* deletion on marker mobility could not be assessed as disruption of the *tar-tsp-cheRBYZ* operon perturbed the number and disposition of fluorescent tags; *tidL* insulation properties were demonstrated by inserting it at an ectopic position (see below).

### *tidR* and *tidL* insulate the NS regions from the Ter macrodomain

**Figure 4:**
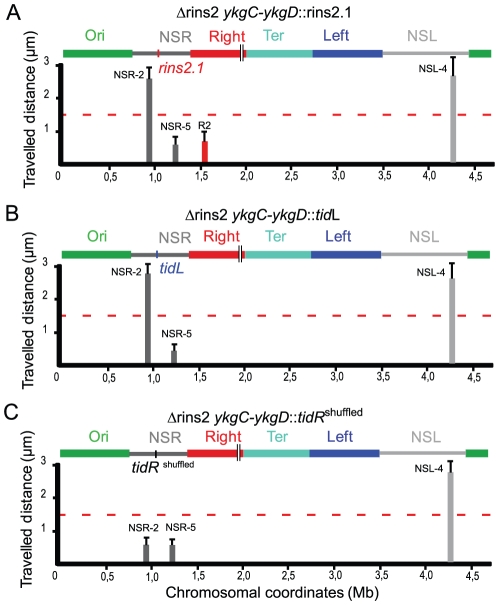
*tid*R and *tid*L insulate the Ter MD. Travelled distance of different markers in strains with a Δrins2 deletion, and carrying segment rins2.1, *tid*L or a control sequence in the middle of the NS^Right^ region. The representation is the same as in Figure 2. (A) strain Δ*rins2* ykgC-ykgD::rins2.1 with a deletion of the segment rins2 and an insertion of segment rins2.1 between *ykgC* and *ykgD* (markers NSR-2, NSR-5, Right-2 (R2), NSL-4 are indicated from left to right). (B) strain Δrins2 *ykgC-ykgD*::*tid*L with a deletion of the segment rins2 and insertion of *tid*L between *ykgC* and *ykgD* (markers NSR-2, NSR-5 and NSL-4 are indicated from left to right). (C) strain Δrins2 *ykgC-ykgD*::*tid*R^shuffled^ with a deletion of the segment rins2 and an insertion of shuffled sequence with a similar palindromic organization as *tid*R between *ykgC* and *ykgD* (markers NSR-2, NSR-5, NSL-4 are indicated from left to right).

To determine whether *tid*R/L could act at another position, the 500 bp fragment carrying *tid*R deleted in strain ΔRins2.1 was inserted in the middle of the NS^Right^ region, between markers NSR-2 and NSR-5, in a Δrins2 strain (Figure 4A). The NSR-5 marker which is closest to the Ter MD showed a decrease in mobility compared to the wild type situation, similar to what was observed in a Δ*tid*R strain. Remarkably, the NSR-2 marker, farthest from the Ter MD, displayed a mobility similar to the wild type situation, in contrast to what was observed in a Δ*tid*R strain. These results indicated that the fragment containing *tid*R protected from the constraining effect originating from the Ter MD at various positions, in the Right MD or in the NS^Right^ region. They suggest that *tid*R works in *cis* by impeding the progression along the chromosome of a process that changes the dynamic properties of the DNA molecule.

To test whether the 12 bp *tidR*/*L* sequence was sufficient to protect from the constraining process, *tidL* was inserted at the same location between markers NSR-2 and NSR-5 (Figure 4B). Insertion of the *tidL* sequence protected the marker NSR-2 from the constraining effect indicating that the 12 bp sequence was sufficient to impede the constraining process (Figure 4B); similar results were observed with the 12 bp *tidR* sequence (data not shown). The effect was specific for *tid*R and *tid*L as a 12 bp control shuffled palindromic sequence GACGCTAGCGTC had no effect on impeding the change of mobility of NSR-2 (Figure 4C). These results allowed the definition of the 12-mer GYTGACGTCAGC consensus sequence that insulated the NS regions from a long range *cis*-acting constraining effect promoted by the Ter MD. *tidR* and *tidL* were the only occurrences of this sequence found in the *E. coli* genome. By bioinformatic analyses, the presence of *tidRL* flanking the Ter MD was found conserved only in *Shigella* species; other enterobacterial genomes lack *tidRL* sequences (e.g. *Yersinia pestis*) or carry multiple copies (e.g. in *Salmonella typhimurium*).

### Insulation of the NS regions from the Ter MD involves a protein co-occurring with the Dam methylase and MatP

**Figure 5:**
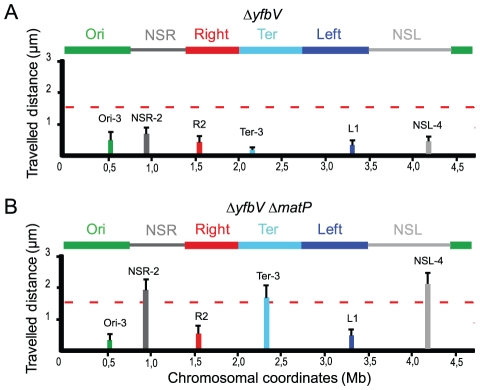
Insulation requires the YfbV protein. Travelled distance of different markers in strains deleted for *yfbV or yfbV and matP*. The representation is the same as in Figure 2. (A) strain with a deletion of *yfbV* (markers Ori-3, NSR-2, Right-2 (R2), Ter-3, Left-1 (L1) and NSL-4 are indicated from left to Right). (B) strain with a deletion of *yfbV* and *matP* (markers Ori-3, NSR-2, Right-2 (R2), Ter-3, Left-1 (L1) and NSL-4 are indicated from left to right).

MatP belongs to a group of 18 proteins exclusively identified in bacteria with Dam methyltransferase activity [14]. We wondered whether one of these proteins might be involved in the insulation mechanism preventing the spreading in *cis* of the constraining process. In order to test this hypothesis, we analyzed the mobility of markers in mutants of each of the genes encoding proteins of unknown function. Strikingly, the mobility of markers located in the NS^Right^ region and in the NS^left^ region was reduced in a *yfbV* mutant, whereas mobility of markers located in the different MDs was not affected (Figure 5A, see Figure 2A for comparison). This protein might thus be implicated in the insulation process promoted by both *tid*R and *tid*L. Interestingly, the *yfbV* gene encodes a predicted protein of 151 amino acids with two internal trans-membrane segments imposing the following topology: a 45 aa N-terminus cytoplasmic domain, a trans-membrane domain composed of two trans-membrane segments separated by a small periplasmic loop of 3 aa, and a 60 aa C-terminal cytoplasmic domain.

The reduction in mobility of markers located in the NS^Right^ region and in the NS^left^ region observed in a *yfbV* mutant was not seen in a *yfbV matP* double mutant (Figure 5B), confirming the involvement of *yfbV* in impeding the spreading of the constraining process originating from the Ter MD.

### The constraining process relies upon the association of the Ter MD with the division machinery

**Figure 6:**
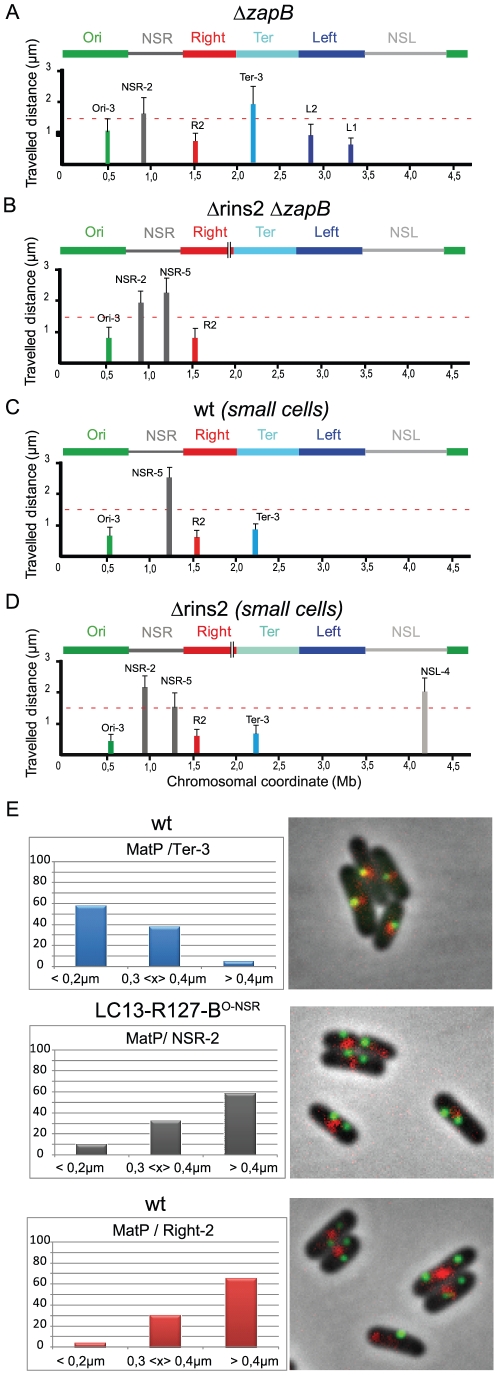
The constraining process depends on MatP and ZapB. (A–D) Travelled distance of different markers in strains deleted for *zapB*, deleted for segment rins2 and *zapB*, and in wt strain. The representation is the same as in Figure 2. (A) strain with a deletion of *zapB* (markers Ori-3, NSR-2, Right-2 (R2), Ter-3, Left-2 (L2), Left-1 (L1) are indicated from left to right). (B) strain with a deletion of *zapB* and rins2 (markers Ori-3, NSR-2, NSR-5, Right-2 (R2) are indicated from left to right). (C) small cells of wt strain before Ter MD segregation at mid-cell (markers Ori-3, NSR-5, Right-2 (R2), Ter-3 are indicated from left to right). (D) small cells of strain Δrins2 before Ter MD segregation at mid-cell (markers Ori-3, NSR-2, NSR-5, Right-2 (R2), Ter-3, NSL-4 are indicated from left to right). (E) Quantification of the co-localisation of the MatP-mCherry focus (red) with a chromosomal *parS* tag bound by ParB-GFP (green). The percentage of cells presenting different interfocal distances is given (left column). In the top lane, the MatP-mCherry focus co-localizes with the Ter-3 marker in the wt strain. The interfocal distance is smaller than 0.4 µm in more than 90% of the cells. In the central lane, the MatP-mCherry focus does not co-localize with the NSR-2 marker in the strain LC13-R127-B^O-NSR^ with a transposition of the Right MD between the Ori MD and the NS^Right^ region (see chromosome configuration in Figure 2). The interfocal distance is greater than 0.3 µm in almost 90% of the cells. In bottom lane, the MatP-mCherry focus does not co-localize with the Right-2 marker in the wt strain. The interfocal distance is greater than 0.3 µm in 90% of the cells.

It was recently shown that the Ter MD is associated with components of the divisome during part of the cell cycle. This association relies on the interaction of MatP with ZapB (Espeli et al., submitted). In order to probe the role of this association in the constraining process, we analysed the mobility of markers in cells devoid of ZapB (Figure 6A). In a *zapB* mutant, the mobility of a Ter marker was increased, whereas mobility of markers located in the other MDs or in the NS regions remained the same (See Figure 2A for comparison). Remarkably, deleting the *tidR* palindrome in this *zapB* mutant context did not lead to a decreased in mobility of makers located in the NS^Right^ region, in contrast to what was observed in a wild type background (Figure 6B, see Figure 3D for comparison).

This suggests that the constraining process affecting the NS regions in the absence of *tidR* and *tidL* may depend upon the association of the Ter MD with the divisome. This interaction lasts for about half of the cell cycle, and mobility of markers was routinely measured during that period. In order to confirm that the constraining process really required this association, we measured the mobility of markers during the part of the cell cycle when the Ter MD did not interact with the divisome, i.e. in small cells in which the Ter MD has not yet been segregated (Figure 6C). In these cells, the mobility of a Ter marker was slightly increased, which was consistent with the fact that association with the divisome added a level of constraint onto the Ter MD (Espéli et al., submitted). Strikingly, a marker in the NS^Right^ region was as mobile in small Δ*tid*R cells as in small wild type cells (Figure 6D).

Altogether, these results demonstrated that the constraining process observed in the NS^Right^ region when *tidR* is absent depends on the association of the Ter MD with the division machinery.

### Spatial separation of the Ter MD territory and of the constrained region

It was previously shown that MatP associates with the entire 800 kb Ter region (Mercier et al., 2008). We wondered whether the constraining process originating from the Ter MD required the recruitment of the constrained NS regions within the Ter MD territory. To test this hypothesis, we analysed the co-localization of different markers with a MatP-mCherry fusion protein (Figure 6E). In the wild type chromosomal configuration, the Ter-2 marker was co-localized with the MatP focus, whereas the Right-2 marker was not (Figure 6E). In a strain where the NS^Right^ region has been transposed next to the Ter region (strain LC13-R127-B^O-NSR^), the NSR-2 marker did not co-localize with the MatP focus as observed for marker Right-2 in the wild type configuration (Figure 6E). These results indicated that constraining of the NS region did not result from a recruitment of the constrained region in the Ter MD territory.

### Long-range DNA interaction of the NS^Right^ region is not affected by the MatP-associated constraining effect

Long distance DNA collisions revealed by the frequency of λ site-specific recombination between λ *att*R and *att*L sites inserted at different chromosomal locations can be used to reveal chromosome conformation [11]. Inactivation of MatP leads to increased collisions between markers in the Right MD and the Ter MD [13]. To assess whether the constraining of the NS^Right^ region upon deletion of *tid*R could affect long range DNA collisions inside the NS^Right^ region or between the NS^Right^ region and the flanking Right MD, interactions between *att*L inserted in the NS^Right^ region and *att*R sites located at different positions in wt or Δrins2 cells. Remarkably, interactions were as frequent in both genetic contexts, either within the NS^Right^ region or between the NS^Right^ region and the Right MD (Table S3).

### Constraining of the NS regions affect their segregation following replication

The organization of the chromosome into macrodomains influences the segregation of sister chromatids and the mobility of chromosomal DNA [12]. Above is described the effect of the absence of the insulation determinants upon DNA mobility. We then examined its impact on the extent of co-localization following replication, by measuring the co-localization index (see Materials and Methods). In the wild type strain, this co-localisation index was high for markers located in MDs (>0.1) in contrast to markers in NS regions (<0.02). Remarkably, only the index of co-localization of the NS^Right^ region marker increased in a Δ*tid*R mutant, whereas it increased for markers present in both the NS^Right^ and NS^Left^ regions in the *yfbV* mutant (Table 2). The increase of co-localization index for markers in NS regions was correlated with a higher number of cells carrying only one focus, localized close to mid-cell.

**Table 2:** Co-localisation index of chromosomal markers.

	WT	Δ*rins2*	Δ*yfbV*
Ori-3	0,15	0,12	0,13
NSR-2	0,01	0,16	0,14
Right-2	0,14	0,16	0,16
Ter-3	0,34	0,33	0,35
NSL-4	0,01	0,02	0,15

## Discussion

### A new method to engineer the bacterial chromosome

In various studies, inversion of chromosomal segments has been used to change gene positioning or chromosome configurations. Although these approaches have been very useful to reveal various features of gene and genome organization, genetic inversions affect several parameters: gene orientation relative to replication, gene dosage through the change of position relative to oriC, disruption of genetic organization at the two sites of recombination. We have devised a new method to rearrange the bacterial chromosome. The experimental design involved two steps, excision of a segment followed by the reinsertion at another location, and the desired chromosomal configuration can be directly detected on plates with a coloured indicator. This method allows the transposition of fragments ranging in size from a few kilobases to hundreds of kilobases. Because the orientation of genes and sequences are conserved, it allows the transposition of any segment on the chromosome; we succeeded in making all the rearrangements we wanted to perform. The only limitation of the method is the requirement for an essential gene in the transposed segment. In the absence of such a gene, all clones that sustained the deletion will grow making difficult the detection of clones that recombined the excised molecule.

### Association of the Ter MD to the division machinery constrains DNA

Markers in the MD and NS regions can be distinguished by their mobility and by the extent of colocalization after replication. The juxtaposition of NS regions to the Ter MD, the deletion of the insulators or the inactivation of YfbV provoked a change of these two properties in the NS regions. Remarkably, the constraining process appeared to spread in *cis* as insertions of the insulators in the middle of the NS^Right^ region insulated the distal part but not the part proximal to the Ter MD. This effect was regulated during the cell cycle as it was promoted by the interaction of the Ter MD with the division machinery at mid-cell.

The spreading of the constraining process can not be visualized in Right and Left MDs of wt cells because of the reduced mobility of markers in these regions. However, in *yihI* mutant cells in which the mobility of markers in the Right and Left MDs is released to a level similar to that of NS regions (Valens et al., in prep), mobility of markers of the Right MD located between the Ter MD and *tidR* were constrained by the MatP-associated constraining process (data not shown). These results indicate that, in the absence of insulation barrier *tidR*, the constraining process affects not only the NS^Right^ region but also the Right MD in agreement with a process spreading in *cis*.

**Figure 7:**
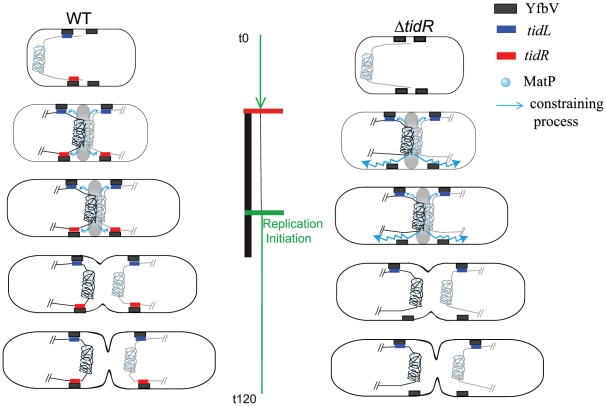
Model for the insulation of the Ter MD by *tid*RL. In wt cells (left panel), the Ter MD (only the Ter MD and the flanking DNA is represented) is found near the new pole. As the cell cycle progresses (see [13] for details), the Ter MD is segregated at mid-cell. Interaction of MatP with ZapB promotes the tight association of the Ter MD with the divisome (represented by a grey oval), period indicated by the vertical black bar. At this stage, the constraining process (schematized by a wavy blue line) is apparent but does not propagate behind *tid*R and *tid*L. The constraining process would abrogate when the Ter MD dissociates from the divisome. The insulation mechanism requires a direct or indirect association of *tidR* with YfbV. It is not known if this association lasts during the entire cell cycle. In Δ*tid*R cells (right panel), insulation is efficient only on the left arm of the chromosome and the constraining process spreads to the right arm of the chromosome. The replication period of wt cells is represented by the green vertical bar; replication is initiated in the mother cell (indicated by a green horizontal bar) and terminates soon after birth (indicated by a horizontal red bar).

It is not yet known how MatP organizes the Ter MD at the molecular level. The association of the Ter MD with the division machinery modifies its properties and probably affects the overall structure of the Ter region; targeting of the Ter MD to the division machinery could promote the formation of loops between *matS* sites via the interaction of MatP with ZapB assembled in the FtsZ ring (Espéli et al., submitted). In the absence of the insulator, the macromolecular complexes assembled at mid-cell provoked a change of DNA properties detectable at several hundreds of kilobases (Figure 7). The change of properties at a long distance might either result from a change at the chromatin level by the binding/tracking of protein(s) or from modifications of physical properties of the DNA molecule. We do not favour the first hypothesis as it is hard to conceive how the effect would be related to the targeting of the Ter region to the division machinery and how binding or tracking of a protein could act in *cis* over a region of several hundreds of kilobases. A plausible hypothesis implies modifications of physical properties of DNA. Using two different topological reporter systems that reveal changes in local DNA supercoiling (expression of a *lacZ* gene under the control of the supercoiling sensitive promoter P_gyrA_ and site-specific resolution reaction between two γδ *res* sites catalyzed by the γδ resolvase [6], [20]), we failed to detect modifications in the topological properties of the DNA molecule in the absence of insulation (data not shown). We must therefore consider a hypothesis involving an affect on other mechanical properties of the DNA molecule. Although the molecular mechanism is not yet identified, we can imagine that it requires two “anchorages” between which DNA is constrained. While the association of the Ter MD to the division apparatus could constitute the first anchorage, the second one would implicate the tethering of other chromosomal regions in the cell. Because the process is effective up to the NS regions, the second anchorage might involve the Ori region. By acting as a mechanical relief, *tidRL* would restrain the constraining process and would restrict it to the Ter region.

A number of processes are known to occur in the terminus of the chromosome; they include replication termination [21], decatenation of entangled chromosomes [22], chromosome dimer resolution [23] and coupling of replication termination with cell cycle progression [15]. It is tempting to speculate that the changes in DNA properties resulting from the association with the division machinery of the Ter region facilitate certain DNA metabolic processes required for the proper segregation of the chromosome. A challenge now is to characterize the molecular architecture of the Ter MD associated to the division machinery, to identify the process(es) occurring at this stage and reveal the contribution of the change of DNA properties for the efficiency of the process(es) involved.

### An insulation system that controls the formation of MDs and NS regions

By genetic [11] and cytological [12] approaches, we have revealed a chromosome organization into structured MDs and less structured NS regions. Our subsequent analyses have revealed the molecular nature of the Ter MD structuring [13]. Here we have identified a new system that is related to the process involved in the structuring of the Ter MD. This system allowed restricting the effects associated to the interaction of the Ter region with the division machinery. Two 12 bp motifs that flank the Ter MD and one protein co-occurent with MatP have been shown to be required for the insulation process.

The two sequences *tid*R and *tid*L were found in the MD flanking the Ter MD, at 130 kb on the right side and at 50 kb on the left side, respectively. This positioning probably reflects the potential of MatP to act at a distance greater than 50 kb [13] that likely precludes the positioning of the insulators closer to the *matS* sites located at the borders of the Ter MD. Remarkably, *tid*R and *tid*L conserved their activity when moved several hundred kilobases away from their original position; their presence close to the Ter MD might thus not rely on specific positional requirements for the insulation activity but rather serve to prevent the spreading of the constraining process to the flanking regions.

*tid*R and *tidL* are the only occurrences of the 12-mer GYTGACGTCAGC consensus sequence in the *E. coli* genome. Allowing one nucleotide difference in the *tid*RL consensus sequence revealed the presence of 34 sites in the entire genome, dispersed in the diverse MD and NS regions (Figure S4). Based on their position on the genetic map and the properties of markers in various strains, 28 were predicted to be non functional for the insulation property. For 6 variants (2 in the Ter MD and 4 in the Ori MD), it was not possible to predict the functionality.

At least two types of insulation mechanisms could be envisioned at this stage. In both cases, the insulator would block the unidirectional progression of the constraining process since it acts in *cis*. In the first type, a nucleoprotein complex formed at the insulator would act as a barrier and block the progression of a factor tracking along the DNA molecule. This process would be reminiscent of that selected in boundary elements that prevent the spreading of heterochromatin via the binding of proteins to specific sequences in yeast or vertebrate cells [24], [25] or of that at work with the Tus/ter system that block replication fork progression in bacteria [26]. The second type may result in barrier function by interfering with the spreading of changes affecting physical properties resulting from the MatP-*matS* complexes. In the last case, anchoring of the DNA molecule to a cellular structure may be required to act as a point of physical relief.

We showed that the gene *yfbV* is required for the insulation properties of *tidR* and *tidL*. It is not yet known how YfbV could promote insulation at *tidRL* sequences; however it is tempting to speculate that the anchorage of YfbV in the membrane might be used to tether the insulators to a fixed structure, e.g. the membrane, within the cell. Further analyses will be required to characterize the role played by YfbV in the insulation process.

## Materials and Methods

### Strains and media

The bacterial strains and plasmids used in this study are listed in Table 1. *E. coli* strains were grown at 30°C in Lennox broth, or in minimal medium A supplemented with 0.12% of casaminoacids and 0.5% of glucose. Antibiotics were added when necessary. The different deletions targeted in the chromosome were performed by the one-step insertion-deletion technique [27]. These deletions were done in strains carrying the plasmid pKD46 or in the strain DY330 [28]. Two cassettes used to perform deletion constructions carried either a chloramphenicol or a rifampicin resistance gene. Both resistance genes are flanked by *frt* sites allowing their subsequent deletion [27]. Deletion coordinates are indicated in Table 1. The deletions were verified by PCR.

### Induction of transposition

The strains used for transposition carried three *att*L, *att*R and *att*B′ sites derived from the λ site-specific integration module. The three *att* sites were inserted in the same orientation. Each site was flanked by a cassette carrying an antibiotic resistance gene (chloramphenicol, kanamycin and apramycin, respectively). In addition, the *att*L site was flanked by the 5′ part of *lacZ* and the *att*R site was flanked by the 3′ part of *lacZ*. The strains are transformed by the plasmid pTSA-CXI that expressed the λ recombinase [11] to promote the transposition. The strains were grown at 30°C and shifted to 39°C to induce the production of the recombinases. After the recombination step, the strains were plated on LB medium supplemented with Xgal and blue colonies were selected. The transposition reactions were verified by PCR verification with appropriate primers (Figure S1).

### Fluorescence microscopy and mobility of DNA markers

Cultures were grown in minimal A medium in the presence of glucose and casaminoacids without IPTG to maintain at a minimal level expression of *gfp-parB* present on plasmid pALA2705 [19]. Movies were recorded automatically on a Leica microscope. Autofocus was performed at every time point on the phase contrast image and GFP fluorescence was recorded on the plane with the best phase contrast focus. Image analysis was performed with ImageJ software using the manual tracking plugin (http://rsb.info.nih.gov/ij/index.html). The XY co-ordinates of the two poles and of the foci were recorded manually and processed automatically with Excel (Microsoft) software. The travelled distance was estimated over a period of 5 minutes by adding up the absolute values of the distances for all 10 sec interval as described before [12]. The (x,y) coordinates of the foci at every time point were recorded and the distance travelled in the 10 s interval calculated. For the mobility measurements at the home position, 30 foci were analyzed (30 cells with one focus for markers in the Ter MD and 15 cells with two foci for markers in other regions of the chromosome). To measure the mobility of markers, the distance travelled by various foci was recorded over a period of 5 minutes with 10 sec intervals when markers were at home position. In the growth conditions used, at home position, markers of Ori, Right and Left MDs as well as markers from NS regions were segregated in the two halves of the cells whereas markers of the Ter MD are found at mid-cell [12].

### Index of colocalisation

This index is calculated by comparing the theoretical number of genes (nb^th^) [29] to the experimental number of genes, i.e. the number of foci detected by microscopy (nb^obs^) [12]. The normalization of the index is obtained by the formula: [(nb^th^−nb^obs^)/nb^th^].

## Supporting Information

Figure S1 PCR reactions to control the transposition reaction generating strain LC13-R127-B^O-NSR^. (A) Schematic map of *att* sites in strain LC13-R127 before and after transposition. To control the result of the transposition reaction, 5 pairs of primers flanking the *att* sites were used (black arrows). Pairs 1 and 2 amplify respectively the *att*B and *att*R sites of the chromosome in its original configuration. Pairs 3, 4 and 5 flanked the *att*R′, *att*L′ and *att*B′ sites in the stain after transposition (details will be given upon request). (B) Gel electophoresis of PCR products generated with the genomic DNA of the LC13- R127-B^O-NSR^ strain before (WT) and after transposition (T).
(EPS)

Figure S2 Physiology of the R127-LC13-B^O-NSR^ transposed strain. (A) Cells were grown until OD 0.2 in LB, fixed and DNA stained with DAPI as described before (Esnault et al., 2007). Percentage of cells in each size category according to their number of nucleoids in the wild type strain (left) and the transposed LC13-R127-B^ONSR^ strain (right). Coloured horizontal bars indicate the percentage of the different types of cells and nucleoids. Cell size is indicated. Green indicates cells containing one and two nucleoids; yellow: cells containing four nucleoids; cyan: cells containing *par*-like nucleoids; red: cells with unsegregated nucleoid. (B) Co-culture competition assay between the wild type strain and the LC13-R127- B^O-NSR^ transposed strain. The strain with the NS^Right^ region transposed was grown in serial co-cultures with the wt strain; the assay is performed in LB at 30°C until 80 generations as described before (Esnault et al., 2007). The ratio of transposed to wt configurations is plotted as a function of the number of generations (3 independent experiments were performed).
(EPS)

Figure S3 Cells and nucleoid distribution in strain Δ*tidR*. (A) Cells were grown until OD 0.2 in LB, fixed and DNA stained with DAPI as described before (Esnault et al., 2007). Percentage of cells in each size category according to their number of nucleoids in the wild type strain (left) and the Δ*tidR* strain (right). Green indicates cells containing one and two nucleoids; yellow: cells containing four nucleoids; cyan: cells containing *par*-like nucleoids; red: cells with unsegregated nucleoid.
(EPS)

Figure S4 Alignment of the 34 *tidRL*-like sequences in the *E. coli* chromosome. They differ from *tidR* by one change at a single position. « + » indicates an insulation effect; «−» indicates no insulation effect; « ? » indicates that the insulation effect is not determined or can not be predicted.
(DOC)

Table S1 Coefficient diffusion of chromosomal markers.
(DOCX)

Table S2 *parS* tags used in this study.
(DOCX)

Table S3 Long range interactions measured by excisive recombination.
(DOC)
