# Supplementary material for: Long-Range Chromosome Organization in E. coli: A Site-Specific System Isolates the Ter Macrodomain
Source: PLoS Genet. 2012 Apr 19;8(4):e1002672. doi: 10.1371/journal.pgen.1002672 (PMC3330122; doi:10.1371/journal.pgen.1002672)
Supplement: Figure S4 — Alignment of the 34 tidRL-like sequences in the E. coli chromosome. They differ from tidR by one change at a single position. « + » indicates an insulation effect; «−» indicates no insulation effect; « ? » indicates that the insulation effect is not determined or can not be predicted. (DOC) [file pgen.1002672.s004.doc]

Figure S4 : E. coli sequences differing of tidRL by one change

| position | séquence *tid*R = GCTGACGTCAGC | MD | Insulation effect^1^ |
| --- | --- | --- | --- |
| 224828 | GCTGTCGTCAGC | NSR | - |
| 664088 | GCTGCCGTCAGC | Right | - |
| 1013162 | TCTGACGTCAGC | Right | - |
| 1133301 | GCTGACGGCAGC | Ter | - |
| 1593106 | GCTAACGTCAGC | Ter | ? |
| 1667432 | GCTGCCGTCAGC | Ter | - |
| 1873831 | GCTGACGACAGC | Ter | - |
| 1913726 | GCTGACGTAAGC | Ter | ? |
| 1970549 | GTTGACGTCAGC | Left (*tid*L) | + |
| 2289939 | GCTGACGCCAGC | Left | - |
| 2328312 | GCTCACGTCAGC | Left | - |
| 2561161 | GCCGACGTCAGC | Left | - |
| 2631998 | GCTGAGGTCAGC | Left | - |
| 2711858 | CCTGACGTCAGC | Left | - |
| 2728109 | GCTGACGACAGC | Left | - |
| 2864945 | GCTGACGTCATC | NSL | - |
| 2955254 | GCTGACATCAGC | NSL | - |
| 3308997 | GCTGCCGTCAGC | NSL | - |
| 3425331 | GCTGACGACAGC | NSL | - |
| 3538116 | GCTCACGTCAGC | NSL | - |
| 3745953 | GCGGACGTCAGC | Ori | ? |
| 3889985 | GCTGGCGTCAGC | Ori | - |
| 3940487 | GCTGTCGTCAGC | Ori | - |
| 4034177 | GCTGTCGTCAGC | Ori | - |
| 4052583 | GCTGACGGCAGC | Ori | - |
| 4074929 | GCTGCCGTCAGC | Ori | - |
| 4165295 | GCTGTCGTCAGC | Ori | - |
| 4177610 | GCTGAAGTCAGC | Ori | ? |
| 4206782 | GCTGTCGTCAGC | Ori | - |
| 4245248 | GCTGACGTCATC | Ori | - |
| 4289408 | GCTGACGTTAGC | Ori | ? |
| 4383656 | GCAGACGTCAGC | Ori | ? |
| 4405129 | GCCGACGTCAGC | Ori | - |
| 4543715 | GCTGACGGCAGC | Ori | - |
| 4596789 | GCTGACGCCAGC | Ori | - |

1 : « + » indicates an insulation effect ; «-» indicates no insulation effect ; « ? » indicates that the insulation effect is not determined or can not be predicted
