# Supplementary material for: Long-Range Chromosome Organization in E. coli: A Site-Specific System Isolates the Ter Macrodomain
Source: PLoS Genet. 2012 Apr 19;8(4):e1002672. doi: 10.1371/journal.pgen.1002672 (PMC3330122; doi:10.1371/journal.pgen.1002672)
Supplement: Table S1 — Coefficient diffusion of chromosomal markers. (DOCX) [file pgen.1002672.s005.docx]

**Table S1**: Coefficient diffusion of chromosomal markers

|  |  | coeff diffusion | |  |
| --- | --- | --- | --- | --- |
|  |  | X | Y | X+Y |
| WT | Ori-3 | 1,00E-04 | 1,20E-04 | 1,10E-04 |
|  | NSR-1 | 3,90E-04 | 3,60E-04 | 3,80E-04 |
|  | NSR-2 | 4,50E-04 | 3,80E-04 | 4,10E-04 |
|  | NSR-5 | 4,60E-04 | 3,90E-04 | 4,20E-04 |
|  | Right-2 | 1,00E-04 | 7,30E-05 | 8,80E-05 |
|  | Right-5 | 9,70E-05 | 9,00E-05 | 9,40E-05 |
|  | Ter-3 | 6,00E-05 | 5,60E-05 | 5,80E-05 |
|  | Left-1 | 1,10E-04 | 9,20E-05 | 1,00E-04 |
|  | NSL-3 | 4,20E-04 | 4,10E-04 | 4,20E-04 |
|  | NSL-4 | 3,70E-04 | 4,10E-04 | 3,90E-04 |
| LC13-R127-B^O-NSR^ | Ori-3 | 1,30E-04 | 1,10E-04 | 1,20E-04 |
|  | NSR-1 | 3,80E-04 | 3,50E-04 | 3,60E-04 |
|  | NSR-2 | 1,20E-04 | 8,70E-05 | 1,00E-04 |
|  | NSR-5 | 1,60E-04 | 1,40E-04 | 1,50E-04 |
|  | Right-2 | 1,20E-04 | 1,50E-04 | 1,40E-04 |
|  | Right-5 | 1,30E-04 | 1,10E-04 | 1,20E-04 |
|  | Ter-3 | 4,50E-05 | 3,50E-05 | 4,00E-05 |
|  | Left-1 | 1,30E-04 | 1,10E-04 | 1,20E-04 |
|  | NSL-3 | 4,00E-04 | 5,20E-04 | 4,60E-04 |
|  | NSL-4 | 4,90E-04 | 4,70E-04 | 4,80E-04 |
| LR146-R124-B^L-T2^ | Ori-3 | 1,40E-04 | 1,60E-04 | 1,50E-04 |
|  | NSR-2 | 6,60E-04 | 4,90E-04 | 5,70E-04 |
|  | Ter-3 | 4,60E-05 | 4,80E-05 | 5,00E-05 |
|  | Left-1 | 1,60E-04 | 1,40E-04 | 1,50E-04 |
|  | NSL-3 | 1,50E-04 | 9,00E-05 | 1,20E-04 |
| LC13-R18^inv^/LR146-R31 | NSR-2 | 1,20E-04 | 1,30E-04 | 1,20E-04 |
|  | NSR-5 | 2,30E-04 | 1,90E-04 | 2,10E-04 |
|  | Right-2 | 1,90E-04 | 1,60E-04 | 1,80E-04 |
|  | Left-1 | 1,90E-04 | 1,80E-04 | 1,80E-04 |
|  | NSL-3 | 1,80E-04 | 1,90E-04 | 1,80E-04 |
