# Supplementary material for: Long-Range Chromosome Organization in E. coli: A Site-Specific System Isolates the Ter Macrodomain
Source: PLoS Genet. 2012 Apr 19;8(4):e1002672. doi: 10.1371/journal.pgen.1002672 (PMC3330122; doi:10.1371/journal.pgen.1002672)
Supplement: Table S2 — parS tags used in this study. (DOCX) [file pgen.1002672.s006.docx]

**Table S2**: *parS* tags used in this study

|  | gene name | position (bp) |
| --- | --- | --- |
| ori-3 | *aidB* | 4413507 |
| NSR-1 | *araC* | 71279 |
| NSR-2 | *crl* | 258144 |
| NSR-5 | *ybbL* | 515143 |
| right-2 | *ybfD* | 738100 |
| right-5 | *ycdN* | 1080438 |
| ter-3 | *ydaA* | 1395706 |
| Left2 | *hisI* | 2095272 |
| left-1 | *yfgE* | 2616013 |
| NSL-3 | *ygeB* | 3010469 |
| NSL-4 | *yhbW* | 3302216 |
