# Supplementary material for: Long-Range Chromosome Organization in E. coli: A Site-Specific System Isolates the Ter Macrodomain
Source: PLoS Genet. 2012 Apr 19;8(4):e1002672. doi: 10.1371/journal.pgen.1002672 (PMC3330122; doi:10.1371/journal.pgen.1002672)
Supplement: Table S3 — Long range interactions measured by excisive recombination. (DOC) [file pgen.1002672.s007.doc]

**Table S3**: Long range interactions measured by excisive recombination

|  | WT | Δ*rins2* |
| --- | --- | --- |
| **NS^Right^-NS^Right^**  LC4 R11 (attL^331520^ - attR^59331^)  LC4 R27 (attL^331520^ – attR^147935^ ) | 20.3 ± 2.5  23.4 ± 1.1 | 22,3 ± 5.2  23,5 ± 2.3 |
| **NS^Right^-Right MD**  LC4-R26 (attL^331520^ – attR^884387^) | 15.7 ± 2.1 | 15.2 ± 2.5 |
